# Addressing the Obesity Epidemic: A Genomics Perspective

**Published:** 2007-03-15

**Authors:** Amy Zlot, Astrid Newell, Kerry Silvey, Kiley Arail

**Affiliations:** Oregon Genetics Program, Oregon Health Services, Oregon Department of Human Services; Oregon Genetics Program, Oregon Department of Human Services, Portland, Oregon; Oregon Genetics Program, Oregon Department of Human Services, Portland, Oregon; Oregon Genetics Program, Oregon Department of Human Services, Portland, Oregon

## Abstract

Genomics is the study of the entire human genome and involves not only studying the actions of single genes but also the interactions of multiple genes with each other and with the environment. This article emphasizes the multifactorial nature of common obesity, which is caused by the interaction of genes, environment, and lifestyle. Individual variation in genes that influence behavior, satiety, and taste suggests that a one-size-fits-all approach to reducing or preventing obesity may be ineffective. Data are not yet available to allow for personalized obesity interventions based on genetic predisposition. However, a genomics approach may provide a useful framework for addressing the obesity epidemic. More research is needed before specific targeted public health interventions that include genomic strategies can be effectively integrated into addressing obesity in public health practice.

## Introduction

Obesity is a multifactorial disorder that reflects complex interactions of genes, environment, and lifestyle. Considering the obesity epidemic from a genomics perspective, which takes into account the effect of all genes in the genome as well as the interactions of those genes with each other and with the environment, has the potential to improve the effectiveness of obesity prevention and intervention strategies.

## Evidence of Genetic Role in Weight Regulation

Numerous family studies indicate a genetic component to weight regulation. Stunkard et al (1) show that body mass index (BMI) is highly correlated in identical twins, even if each twin is raised in a different environment. Studies of eating behavior in fraternal and identical twin pairs suggest that a significant portion (45%–60%) of eating behaviors (e.g., binge eating) was due to genetic factors (2).

Studies of children and their parents also point to a genetic role for obesity. Whitaker et al (3) reported that, although not all obese children have obese parents, parental obesity is a significant predictor of children's obesity. Parental obesity more than doubled the risk of obesity for children younger than 10 years. One prospective longitudinal study (4) found that the strongest predictor of obesity was parental obesity, independent of diet or activity. Another study (5) found that, after controlling for environmental factors, children of obese parents had a higher preference for fatty foods, a lower liking for vegetables, a greater tendency to overeat, and a stronger preference for sedentary activities than did children of normal-weight parents.

## How Much of Obesity is Genetic?

Separating genetic factors from other factors is difficult because genes are part of a dynamic system that is constantly in flux in response to environmental cues. Nonetheless, some researchers tried to pinpoint the proportion of obesity attributable to genetic factors as opposed to environmental factors (e.g., sidewalks, community design) or lifestyle factors (e.g., diet, physical activity).

Loos et al (6) reviewed genetic epidemiology studies and concluded that the heritability (proportion of a trait due to genetic factors but not necessarily single genes) of human adiposity is between 30% and 70%, with the highest heritability estimates coming from studies of twins. The risk of obesity (≥90th^th^ BMI percentile) is two to three times higher for a person with a family history of obesity than for a person without such history. This risk increases as severity of family obesity increases. Risk of extreme obesity (BMI > 45) is seven to eight times higher for members of extremely obese families than for members of normal-weight families.

On the basis of their review, Loos et al suggest four levels of genetic contribution to obesity, two of which are affected by whether one lives in an obesigenic environment (i.e., an environment that promotes high caloric intake and low physical activity)On the basis of their review, Loos et al suggest four levels of genetic contribution to obesity, two of which are affected by whether one lives in an obesigenic environment (i.e., an environment that promotes high caloric intake and low physical activity). The four levels are 1) genetic obesity — genetic mutation in a single gene leads to obesity despite environment (1%–5% of cases); 2) strong predisposition ― overweight in nonobesigenic environment and obese in obesigenic environment; 3) slight predisposition ― normal weight in nonobesigenic environment and overweight in obesigenic environment; and 4) genetically resistant ― normal weight in obesigenic environment.

## Specific Genes Associated with Obesity

Although a family history of obesity is a strong predictor of the condition, only 1% to 5% of obesity cases can be explained by a single gene mutation (6). Common obesity (which affects most obese people) is a complex disorder with contributions from multiple genes and gene variants. However, the search for specific genes associated with obesity provides a foundation for understanding the effect of environmental and lifestyle factors on the development of obesity. Mounting evidence suggests that genetic factors are involved in all aspects of weight regulation, including food intake and energy expenditure. Hunger or appetite, eating behavior (e.g., binge eating), taste, satiety, spontaneous physical activity (e.g., fidgeting), metabolic rate, thermogenesis, and motivation to exercise all appear to have genetic correlates. Most single-gene disorders identified to date are associated with mutations in genes that regulate appetite (7).

The list of genes with a possible role in weight regulation is large; more than 400 genes or markers are associated with obesity (8). Some of the more commonly discussed genes and their characteristics are listed in the Table.

## Interactions

Some genetic research gives us a glimpse of the complex relationships between gene variants, genes and age, and genes and environment.

### Gene–gene interactions

Ochoa et al (11) found a significant interaction between two gene variants, PPAR λ 2 and ADR β 3, in the risk of obesity in children and adolescents. After adjusting for family history of obesity, the researchers found that carriers of both gene variants were almost 20 times more likely to be obese than noncarriers (odds ration [OR] 19.5; 95% confidence interval [CI], 2.4–146.8), suggesting a synergistic effect between the two genes.

### Gene–age interactions

The effects of genes may vary depending on a person's age. Argyropoulos et al (12) reported an association between a mutation in the gene for agouti-related protein (a strong appetite stimulator) and obesity in older adults. The mutation was not associated with obesity in study subjects with a mean age of 25 years but was significantly associated with fat and abdominal adiposity in the subjects' parents, whose mean age was 53 years*.*

### Gene–environment–lifestyle interactions

Numerous studies explored gene–environment–lifestyle interactions. Martinez et al (13) found that an interaction between diet and specific genes may affect obesity risk. They found an increased obesity risk for women with a Glu27 variant and a diet with more than 49% of calories coming from carbohydrates. An alternate variant of that same gene was not associated with an increased obesity risk in relation to carbohydrate intake, given the same number of calories consumed. Another study (14) found that moderate alcohol intake was associated with reduced abdominal fat among middle-aged women genetically predisposed to obesity, whereas abstinence or light alcohol intake was not. The study also found that among people with high polyunsaturated fat intake, those with low genetic risk for obesity have lower levels of abdominal fat than those with high genetic risk for obesity. This finding suggests that the effect of diet on obesity depends on genetic factors.

Changes in gene expression that result from epigenetic influences (modifications of DNA structure rather than DNA sequence) were also explored because of their potential role in obesity and associated chronic diseases. Associations between fetal environment (undernutrition or exposure to maternal hyperglycemia) and adult-onset obesity may be due to epigenetic influences that promote fat storage, but possible mechanisms are not well understood (15,16).

### Genes, obesity, and metabolic disorders

Distribution of fat, rather than amount of total body fat, appears to play a key role in metabolic consequences associated with obesity. Excess central abdominal fat is particularly associated with adverse consequences. People with fat concentrated in the abdomen are more likely to develop diabetes than are people with the same amount of fat distributed throughout the body (17). A study of postmenopausal women also suggests that genetics has a role in abdominal fat deposition (18).

## Genomics in the Prevention and Management of Obesity

### Obesity prevention programs

Although many researchers and policy makers recognize the need to mitigate the increasing rate of obesity, studies indicate that the effectiveness of prevention strategies is limited (19). Understanding the biology of weight regulation, including the effect of an obesigenic environment on gene expression, is essential to the development of effective interventions. For instance, if obesity is related to genetic changes caused by fetal exposures in utero or shortly after birth, more emphasis on maternal and infant nutrition will be needed.

As discussed earlier, children with obese parents are at particular risk of becoming obese. Studies are needed to determine the effectiveness of obesity screening and prevention strategies based on family history. Also needed is an evaluation of the effectiveness of identifying children at risk for obesity at an early age (or even prenatally) and of intervening with families to prevent those children from gaining excess weight.

### Nutrigenomics for weight management

The premise of nutrigenomics is that a person's optimal diet is determined by genetic makeup. Nutrigenomics proponents maintain that dietary intervention based on individual genotype can prevent or treat chronic disease and manage weight (20). Although nutrigenomic profiling combined with personalized interventions based on those profiles may help prevent and manage obesity in the future, data to support the efficacy of this approach are not available now (21,22). Unfortunately, lack of data has not stopped companies from marketing genetic tests directly to the public and from making claims about the efficacy of basing nutritional advice on the results of those tests.

### Pharmacogenomics in the management of obesity

Although development of drugs for obesity may have little appeal for those who primarily advocate environmental and lifestyle change, effective medications may be the best hope for some individuals struggling to maintain a healthy weight. Studies to identify the genomics of obesity and use the results to develop drug treatments are under way (23). Aitman (24) suggests that gene expression patterns may help define particular subtypes of obesity and may help in developing antiobesity drugs.

### Environmental approaches

Although gene-based approaches (e.g., nutrigenomics, pharmacogenomics) are not yet ready for widespread application, information from genomic studies gives us clues about how to manage obesity. In 2003, Lowe (25) reported that people with a genetic predisposition to obesity have difficulty self-regulating food intake to maintain weight loss or prevent weight gain, even if they had extensive education in how to do so. Lowe concluded that weight control programs that focus on lifestyle modifications based primarily on cognitive–behavioral change are not effective because they do not consider that availability of food is a biological stimulus to eating, particularly for those genetically prone to obesity. Programs that provide portion-controlled, nutrient-dense meals may be more effective than other programs because they limit opportunities for participants to consume energy-dense foods. Lowe argues that, to stem the tide of obesity, individuals and communities need to eliminate many food stimuli (e.g., overabundance of food, large portion sizes, high-calorie foods).

## Obesity and Future Public Health Practices

Genomics has the potential to improve the ability of public health professionals to address the obesity epidemic. However, additional work is needed to improve our understanding of the role of genes in obesity and to learn how to use that understanding to prevent or manage the condition. Below are specific suggestions.

### Surveillance and epidemiologic investigations

Baseline data are needed to understand familial risk for obesity and to monitor changes in eating behavior. Using family history information to estimate population risk for obesity is a promising concept because family history reflects genetic predisposition, behavioral factors, and shared environmental factors that may contribute to obesity. Identifying population subgroups at greater than average risk of obesity because of familial factors may provide a basis for targeted intervention. More data are also needed on the interactions between genetic risks and environmental and lifestyle risks. Valuable information could be gleaned by adding questions about family history of obesity to population surveys (e.g., Behavioral Risk Factor Surveillance System) and by cross-tabulating family history factors with lifestyle and environmental factors.

### Policy development

The advent of genetic technology introduced a new set of public policy issues. Two such issues are genetic testing and population databases.

**Genetic testing**

Direct-to-consumer marketing of genetic profiling raises many questions about public policy: Should genetic testing be regulated and by whom? Are there potential or real harms to the public from not regulating genetic testing? In the absence of regulation, should public health agencies monitor genetic testing to ensure that public health is not jeopardized?

**Databases**

Large-scale databases containing genetic information could provide information about links between gene variants and health conditions such as obesity. Although the potential benefits to various populations are great, the associated risks for individuals with deleterious genetic variants may also be great. The question is this: how do we protect information in databases so as to prevent discrimination against individuals?

### Ensuring access to health care

A key public health function is to ensure that everyone has access to the services needed to achieve optimal health. The potential for developing genetic tests with results that can be used to decide which drugs are most effective for treating obesity and other conditions holds promise. Pharmacogenomics is already used to treat some diseases (e.g., cancer). However, advances in pharmacogenomics could also aggravate health disparities. As new treatments emerge, public health will have an important role in ensuring fair distribution of services to the entire population (23,24).

### Health education

Despite increasing exposure to genetic information through the media and in schools, the public's genetic literacy remains low. Misperceptions (e.g., genes equal destiny) are common. Public health has an important role in demystifying genetics and clarifying genetic concepts, not only as they apply to obesity but also as they apply to other health conditions. Studies to determine the value of incorporating genomic information into obesity prevention and education programs are needed.

### Professional development

Although health care and public health professionals are working to increase their understanding of genomics, many have limited knowledge of genomic concepts. To use genomics effectively in developing and implementing obesity interventions, public health professionals must increase their understanding of the role of genes in health and disease.

### Public health research

Further research is needed to identify effective individual and population approaches to weight management that incorporate genomic information, and large longitudinal studies are needed to examine gene–environment–lifestyle interactions on a population level (26,27). Such studies must include both sexes, all races and ethnic groups, and people of various ages. Well-executed studies of prenatal subjects and public health populations (e.g., women enrolled in WIC, the U.S. Department of Agriculture's supplemental food program for women, infants, and children) would contribute much to our understanding of genetic risk and its role in preventing or managing obesity. Although scientists hope that personalized health care based on genetic profiling will help people recognize their risk and improve their behavior, additional evidence is needed to support this possibility.

## Research Questions

Although the list of research questions is endless, several come immediately to mind: Can a family history of obesity or genetic screening be used to identify high-risk families and target interventions to specific groups, particularly children? What are the most effective ways of using family history to screen for obesity? What are the most effective intervention programs to prevent weight gain and maintain healthy weight among children from obese families? Does knowing they are at increased risk for obesity (through family history or genetic testing) motivate people to change behavior?

## Conclusions

Obesity is almost always due to a combination of genetic predisposition, lifestyle, and environment. Individual variation in genes that influence eating behavior, satiety, and taste are likely to affect the success of interventions, which suggests that a one-size-fits-all approach may be ineffective in preventing or treating obesity. In addition, because of variations in genetic predisposition, the intensity of interventions required to prevent or treat obesity is likely to vary among individuals. More research is needed to effectively incorporate genomics into public health interventions for obesity.

## Figures and Tables

**Table:** Examples of Genes Involved in Obesity and Their Associated Phenotypes

Gene	Associated Phenotype (Characteristic)
Leptin	Satiation, metabolism
Melanocortin	Feeding behavior, binge eating
Ghrelin	Appetite stimulation
Neuromedin β	Feeding behavior, satiety
PROP	Taste preference
PPAR	Fat metabolism
Mitochondrial uncoupling proteins	Energy expenditure
Melanocortin and MC4R	Energy expenditure

For detailed information about single-gene mutations and their association with obesity, see the *Obesity Gene Map Database* (9) and CDC's *Obesity and Genetics: A Public Health Perspective* (10).
